# Detection rate of brain MR and MR angiography for neuroimaging abnormality in patients with newly diagnosed left-sided infective endocarditis

**DOI:** 10.1038/s41598-023-44253-w

**Published:** 2023-10-10

**Authors:** Seongken Kim, Chong Hyun Suh, Tae Oh Kim, Kyung Won Kim, Hwon Heo, Woo Hyun Shim, Sang Joon Kim, Seung-Ah Lee

**Affiliations:** 1 Department of Radiology and Research Institute of Radiology, Asan Medical Center, University of Ulsan College of Medicine, Seoul, Republic of Korea; 2 Department of Cardiology, Asan Medical Center, University of Ulsan College of Medicine, Seoul, Republic of Korea

**Keywords:** Infectious diseases, Neurological disorders

## Abstract

We aimed to investigate the detection rate of brain MR and MR angiography for neuroimaging abnormality in newly diagnosed left-sided infective endocarditis patients with/without neurological symptoms. This retrospective study included consecutive patients with definite or possible left-sided infective endocarditis according to the modified Duke criteria who underwent brain MRI and MR angiography between March 2015 and October 2020. The detection rate for neuroimaging abnormality on MRI was defined as the number of patients with positive brain MRI findings divided by the number of patients with left-sided infective endocarditis. Positive imaging findings included acute ischemic lesions, cerebral microbleeds, hemorrhagic lesions, and infectious aneurysms. In addition, aneurysm rupture rate and median period to aneurysm rupture were evaluated on follow-up studies. A total 115 patients (mean age: 55 years ± 19; 65 men) were included. The detection rate for neuroimaging abnormality was 77% (89/115). The detection rate in patients without neurological symptoms was 70% (56/80). Acute ischemic lesions, cerebral microbleeds, and hemorrhagic lesions including superficial siderosis and intracranial hemorrhage were detected on MRI in 56% (64/115), 57% (66/115), and 20% (23/115) of patients, respectively. In particular, infectious aneurysms were detected on MR angiography in 3% of patients (4/115), but MR angiography in 5 patients (4.3%) was insignificant for infectious aneurysm, which were detected using CT angiography (n = 3) and digital subtraction angiography (n = 2) during follow-up. Among the 9 infectious aneurysm patients, aneurysm rupture occurred in 4 (44%), with a median period of aneurysm rupture of 5 days. The detection rate of brain MRI for neuroimaging abnormality in newly diagnosed left-sided infective endocarditis patients was high (77%), even without neurological symptoms (70%).

## Introduction

Infective endocarditis is a rare, life-threatening disease, and its neurological complications show the high prevalence and prognostic significance^1–4^. In patients with infective endocarditis, the most frequent complications are neurological and have been reported in 13–55% of cases, and a considerable number of these complications are clinically silent^5–7^. Furthermore, these complications are closely associated with poor prognosis^2,4,8^. Given the high mortality rate and clinically silent occurrence, the management, including brain imaging, of neurological complications should be prioritized in patients with infective endocarditis^9^.

The 2015 European Society of Cardiology guidelines recommend brain imaging when neurological complications are suspected^10^. The minor criteria of the modified Duke criteria, which are widely employed to diagnose infective endocarditis, include positive brain magnetic resonance imaging (MRI) findings, including embolic infarcts, infectious aneurysms, and intracranial hemorrhages^10^. In contrast, the guidelines issued by the American Heart Association (AHA) mention the lack of well-established indications for the routine use of brain MRI and MR angiography in infective endocarditis and the controversial question whether brain MRI for the detection of neuroimaging abnormalities should be performed in all patients with infective endocarditis^11^. Infectious aneurysm is one of the most dangerous neuroimaging abnormalities in infective endocarditis, but is difficult to diagnose and relatively few studies have investigated infectious aneurysm despite its known risk potential^12^.

Despite the lack of evidence on the indications for routine use of brain MRI in patients with left-sided infective endocarditis without neurological symptoms, the AHA guidelines state that cerebrovascular imaging, such as brain MRI, may be considered to rule out metastatic foci of infection and intracranial infectious aneurysms in all patients with left-sided infective endocarditis without neurological symptoms^11^. We hypothesized that brain MRI reveals a high proportion for detecting neuroimaging abnormalities in patients with left-sided infective endocarditis, even without neurological symptoms. This study was conducted with an aim to evaluate the rates of detection of neuroimaging abnormalities with brain MR and MR angiography in patients with newly diagnosed left-sided infective endocarditis, with or without neurological symptoms, and to investigate the detection rate of infectious aneurysm on MR angiography.

## Materials and methods

Asan Medical Center Institutional Review Board (IRB) approved this retrospective, single-institution study. The requirement for informed consent from the study subjects was waived by the IRB of Asan Medical Center due to retrospective study design. All methods were performed in accordance with the Declaration of Helsinki. The datasets used and/or analyzed during the current study available from the corresponding author on reasonable request.

### Patients

**Figure 1:**
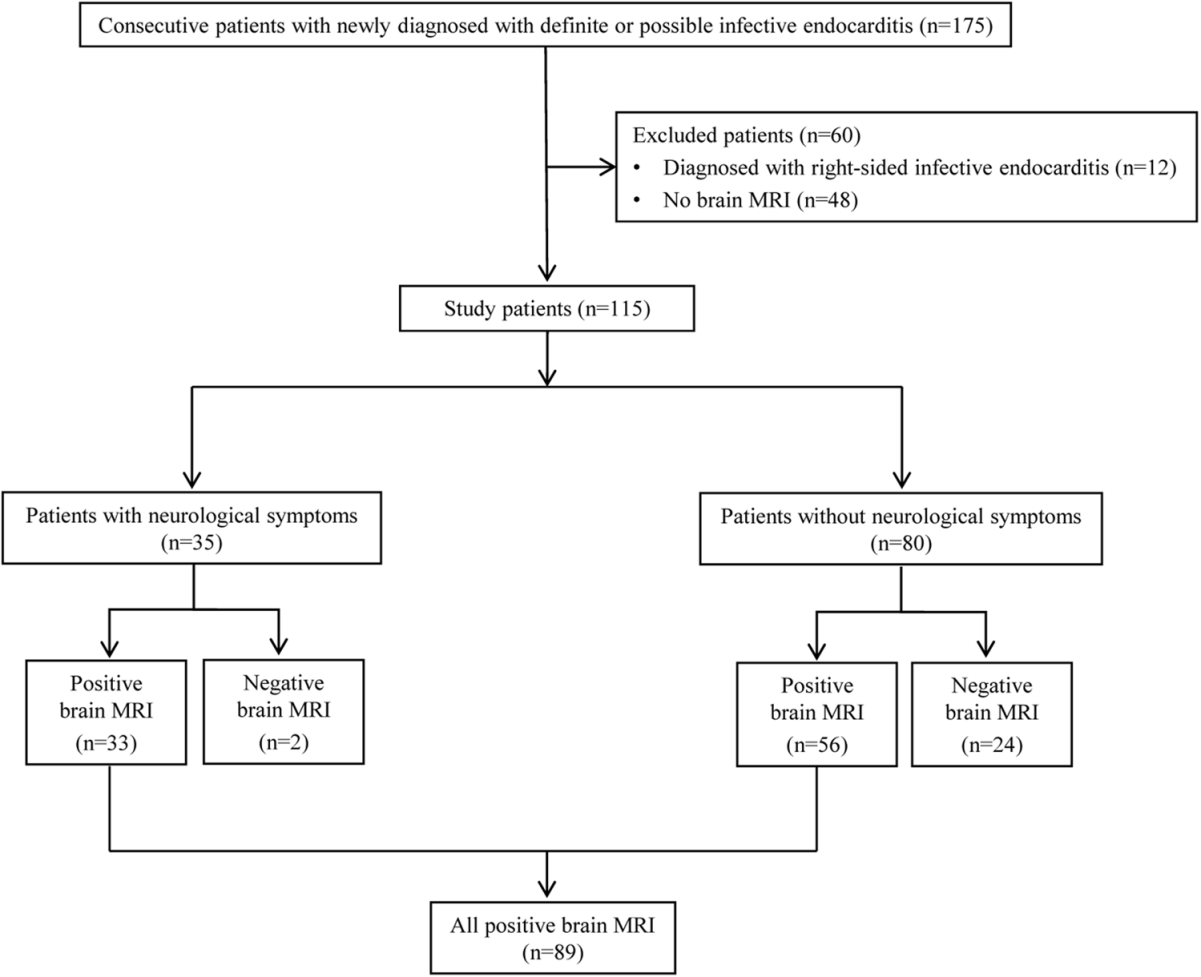
Patient flow chart.

Of the 175 consecutive patients who were newly diagnosed with definite or possible infective endocarditis according to the modified Duke criteria (2015 ESC guideline^10^) between March 2015 and October 2020 in a tertiary referral hospital, we selected participants based on the following eligibility criteria: (a) diagnosis of left-sided infective endocarditis, and (b) underwent brain MRI and MR angiography as an initial evaluation before the diagnosis of infective endocarditis. After history-taking, complete physical–neurological examination, and systemic work-up, including echocardiography, 12 of the 175 patients were diagnosed with right-sided infective endocarditis. Moreover, 48 patients who did not undergo brain MRI were excluded for the following reasons: (1) hemodynamically unstable state; (2) implantable cardioverter defibrillator insertion; (3) incompatible permanent pacemaker insertion; and (4) use of an external hospital brain MRI. Finally, a total of 115 participants were enrolled (Fig. 1) and the neurological symptoms of all patients were assessed at the timepoint when the brain MRI was performed.

Each participant’s medical records and blood culture, echocardiogram, and pathology reports were reviewed independently by a cardiologist (S. L. with 8 years of experience in cardiology), and the participant was classified as having definite infective endocarditis, possible infective endocarditis, or rejected infective endocarditis, according to the modified Duke criteria. Accordingly, 108 patients were diagnosed with definite infective endocarditis as follows: (a) 52 patients who met the pathological criteria (80 of 124 patients underwent cardiac surgery for infective endocarditis); (b) 52 patients who met two of the modified Duke major criteria, including positive blood cultures and positive echocardiogram or CT imaging findings; and (c) 4 patients who met one major and three modified Duke minor criteria. Seven patients were diagnosed with possible infective endocarditis and met one major and one or two modified Duke minor criteria. Patients who were confirmatively diagnosed with an alternative disease, or did not meet the criteria for definite or possible infective endocarditis, were classified as those with rejected infective endocarditis. Additionally, 38 patients were diagnosed with prosthetic valvular endocarditis.

### MRI protocol

Our institutional protocol includes brain MRI and MR angiography for all patients with newly diagnosed definite or possible left-sided infective endocarditis. Specifically, brain MRI using the 3.0-T systems (Ingenia; Philips Healthcare, Architect; GE Healthcare, and Skyra; Simens Medical Solutions) was performed with the following protocol: sagittal T1-weighted imaging, axial T2-weighted imaging, axial fluid-attenuated inversion recovery imaging, axial non-enhanced T1-weighted imaging, axial gradient echo imaging, axial diffusion-weighted imaging, contrast-enhanced 3D T1-weighted imaging obtained in the sagittal plane and reconstructed in the axial and coronal planes, 3D intracranial TOF MR angiography, and contrast-enhanced MR angiography. The details of the MRI protocol are provided in the Supplementary Table e-1–e-3.

### MRI analysis

All brain MRI results were reviewed, wherein it was deemed positive when the specific findings included: acute ischemic lesions, cerebral microbleeds, hemorrhagic lesions, infectious aneurysms, abscess, meningoencephalitis, and ventriculitis. Acute ischemic lesions were defined as high-signal-intensity lesions on T2-weighted imaging with a corresponding diffusion restriction on diffusion-weighted imaging^13^. Cerebral microbleeds were defined as low-signal-intensity lesions of less than 1 cm that were seen on T2*-weighted imaging^14^. Hypertensive and preceding microbleeds were excluded, and we evaluated the remaining microbleeds. Based on their typical locations (basal ganglia, thalamus, and pons), we excluded hypertensive microbleeds^15^. Moreover, we evaluated changes in the microbleeds of patients who underwent follow-up MRI. Hemorrhagic lesions included superficial siderosis, intracranial hemorrhage (ICH), subarachnoid hemorrhage (SAH), and subdural hemorrhage (SDH)^16^, which were defined as lesions detected on T1-weighted, T2*-weighted and fluid-attenuated inversion recovery imaging^17^. Also, convexity SAH was also investigated when we evaluated SAH^18^. Compared to general intracranial aneurysms, aneurysms that were peripherally located and had a fusiform shape, irregular outline, and multiplicity were defined as infectious aneurysms^19^. Brain MRI and MR angiography including contrast-enhanced 3D sequence, T2*-weighted sequence, and fluid-attenuated inversion recovery sequence were performed to detect infectious aneurysms^20–22^. Further studies, including CT and digital subtraction angiographies, were performed in not only patients with infectious aneurysm detected on brain MRI but also those with cerebral hemorrhage and suspected infectious aneurysm undetected on brain MRI. To assess the risk of an infectious aneurysm, the aneurysmal rupture rate and median period to aneurysmal rupture were also evaluated. Abscess^23^ and meningoencephalitis were defined as post-contrast rim-enhancing lesions with central diffusion restriction on diffusion-weighted image as well as sulcal hyperintensity on fluid-attenuated inversion recovery imaging with corresponding post-contrast leptomeningeal enhancement^24^.

All MRI, including both positive and negative brain MRIs, were reviewed by two radiologists based on consensus (S. K., with 2 years of experience in general radiology, and C. H. S., with 10 years of experience in neuroradiology) who were blinded to the clinical information.

### Clinical data and study outcomes

Clinical data recorded after the diagnosis of left-sided infective endocarditis were obtained through a review of medical records. Data on the neurological manifestations, neurological interventional or surgical outcomes, and in-hospital mortality were extracted from the medical records. The primary outcome was the detection rate of neuroimaging abnormality on brain MRI, which was defined as the number of patients with positive brain MRI divided by the number of patients with definite or possible left-sided infective endocarditis. Subgroup analyses of patients diagnosed with definite infective endocarditis were conducted.

### Statistical analysis

The primary outcome was measured with the exact 95% confidence intervals (CIs), and a chi-square test was performed to compare the detection rate for neuroimaging abnormalities between patients with and without neurological symptoms. Statistical analysis was performed using the MedCalc software (Version 19.1; MedCalc Software, Mariakerke, Belgium), and statistical significance was set at *P* < 0.05.

## Results

### Patient characteristics

Patient characteristics are summarized in Table 1. A total of 115 patients (mean age: 55 years ± 19 [standard deviation]; 65 men) were included in this study; of these, 89 (mean age: 56 years ± 19; 49 men) had positive findings on MRI. In the cohort of 115 patients, 35 (30%, 35 of 115) showed neurological symptoms, including weakness or numbness, aphasia or difficulty in talking, decreased levels of consciousness, headaches, visual abnormalities, and others (Table 1). Fourteen patients died in hospital (12%, 14 of 115), of whom 2 (14%, 2 of 14) died of neurological complications arising from intracranial hemorrhage secondary to infectious aneurysmal rupture.

**Table 1:** Characteristics of patients.

Parameter	All patients (n = 115)
Mean age (years)	55 ± 19
Sex	
M	65 (56.5%)
F	50 (43.5%)
Cardiac surgery	84/115 (73.0%)
Neurological symptom	35/115 (30.4%)
Weakness or numbness	4 (3.5%)
Aphasia or difficulty in talking	5 (4.3%)
Decreased level of consciousness	14 (12.2%)
Headache	6 (5.2%)
Visual abnormalities	2 (1.7%)
Others*	4 (3.5%)
None	80 (69.5%)
Neurologic intervention or surgery	11/115 (9.6%)
In-hospital death	14/115 (12.2%)
Neurological death	2/115 (1.7%)

Others include dizziness, seizure, cognitive impairment, and personality change.

### Neuroimaging abnormality of left-sided infective endocarditis

Among the 115 patients who were newly diagnosed with definite or possible left-sided infective endocarditis, the brain MRI revealed positive findings in 89. The detection rate for neuroimaging abnormality on brain MRI in patients with definite or possible left-sided infective endocarditis was 77% (89 of 115; 95% CI 69–84%). The detection rate of neuroimaging abnormality on brain MRI in patients with left-sided infective endocarditis, with or without neurological symptoms were 94% (33 of 35; 95% CI 80–99%) and 70% (56 of 80; 95% CI 59–79%), respectively. Compared to those without neurological symptoms, patients with left-sided infective endocarditis and neurological symptoms had a significantly higher detection rate of neuroimaging abnormality (*P* = 0.041). All 14 patients who died in-hospital had positive brain MRI findings whereas no patient without any imaging abnormalities died in hospital. The rates of detection of neuroimaging abnormality are summarized in Table 2.

**Table 2:** The detection rate of brain MRI and MRA for neuroimaging abnormality in patients with left-sided infective endocarditis.

MRI findings	All patients (n = 115)	Patients with neurological symptoms (n = 35)	Patients without neurological symptoms (n = 80)	P value
Detection rate for neuroimaging abnormality	89/115 (77%)	33/35 (94%)	56/80 (70%)	0.041
Acute ischemic lesion	64/115 (56%)	22/35 (63%)	42/80 (53%)	0.304
Microbleeds	66/115 (57%)	23/35 (66%)	43/80 (54%)	0.233
Follow up MRI (+)	24/66	9/23	15/43	
No change	14/24 (58%)	5/9 (56%)	9/15 (60%)	
Increased	10/24 (42%)	4/9 (44%)	6/15 (40%)	
Hemorrhagic lesion	23/115 (20%)	12/35 (34%)	11/80 (14%)	0.011
ICH	5/23	2/12	3/11	
SAH	8/23	2/12	6/11	
ICH + SAH	5/23	4/12	1/11	
SDH	1/23	1/12	0/11	
SAH + SDH	1/23	1/12	0/11	
Superficial siderosis	3/23	2/12	1/11	
Infectious aneurysm	4/115 (3%)	0/35 (0%)	4/80 (5%)	0.178
Abscess, meningoencephalitis, ventriculitis	8/115 (7%)	7/35 (20%)	1/80 (1%)	< 0.001

*MRI* magnetic resonance imaging, *ICH* intracranial hemorrhage, *SAH* subarachnoid hemorrhage, *SDH* subdural hemorrhage.

*Statistical significance was set at *P* < 0.05.

Notably, on MRI, acute ischemic lesions were detected in 56% (64 of 115) of all patients, in 63% (22 of 35) of the patients with neurological symptoms, and in 53% (42 of 80) of the patients without neurological symptoms. No significant differences in acute ischemic lesion were noted between patients with and without neurological symptoms (*P* = 0.304).

**Figure 2:**
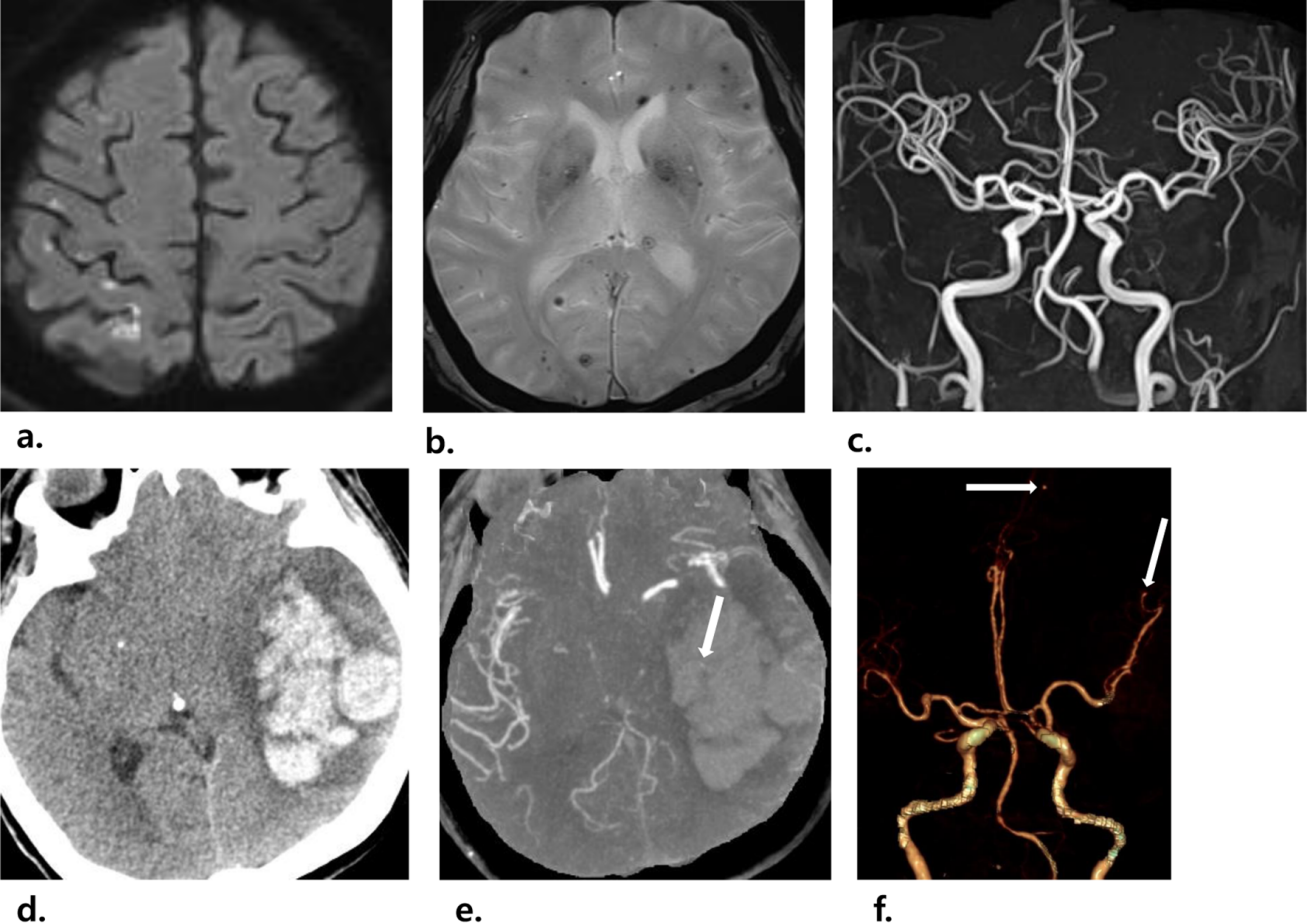
Images of a 69-year-old woman with left-sided infective endocarditis. **(a,b)** Brain MRI images show multifocal, small, high-signal-intensity lesions in the right frontal and parietal lobes on the diffusion-weighted image **(a)**, which suggests embolic infarctions, as well as multiple cerebral microbleeds in both cerebral hemispheres on the T2*-weighted image **(b)**. **(c)** No definite vascular abnormality was detected on brain MR angiography. After 4 days, the patient had drowsy mentality and vomiting, and a CT scan was performed. **(d)** Axial non-contrast-enhanced CT imaging shows a new acute intracranial hemorrhage in the left temporoparietal lobe. **(e)** A small enhancing lesion (arrow in **e**) is seen on axial maximum-intensity projection imaging, suggesting that a infectious aneurysmal rupture caused an acute intracranial hemorrhage. **(f)** On the 3D volume-rendered image of CT angiography, two additional infectious aneurysms were detected in the distal branch of the left ACA and left MCA (arrows in **f**). Despite emergency decompressive craniectomy, the patient died.

Cerebral microbleeds were detected in 57% (66 of 115) of all patients, in 66% (23 of 35) of the patients with neurological symptoms, and in 54% (43 of 80) of the patients without neurological symptoms. Of the 66 patients with microbleeds, 24 underwent follow-up brain MRI, and 10 (42%, 10 of 24) showed an increasing pattern. Microbleeds mainly showed an increasing pattern in deep/subcortical white matter or the cerebellum than in the basal ganglia or thalamus. No significant differences in microbleeds were noted between patients with and without neurological symptoms (*P* = 0.233, respectively). Figure 2 shows images of a patient with left-sided infective endocarditis and embolic infarctions and cerebral microbleeds.

Hemorrhagic lesions were detected on MRI in 20% (23 of 115) of patients of which 19 had ICH and/or SAH on MRI, among which 4 had infectious aneurysms. Of the 14 patients with SAH, 12 patients (86%) appeared to have convexity SAH, and infectious aneurysm was detected in 2 of these patients. Abscess, meningoencephalitis, and ventriculitis were detected on MRI in 7% (8 of 115) of patients of whom 5, 2, and 1 had abscess, meningoencephalitis, and ventriculitis, respectively. The group of patients with neurological symptoms had a significantly higher incidence of hemorrhagic lesions and abscesses, meningoencephalitis, and ventriculitis than that without neurological symptoms (*P* = 0.011 and *P* < 0.001, respectively). Supplementary Figure e-1 shows images of patient with left-sided infective endocarditis and an abscess.

### Infectious aneurysm

Infectious aneurysms were found in 9 patients (7.8%, 9 of 115) and were detected on MR angiography in 4 patients (3%, 4 of 115), but undetected on initial MR angiography in 5 patients. All 4 patients with MR angiography-detected aneurysms had no neurological symptom whereas 5 patients with aneurysms undetected on MR angiography presented neurological symptoms (4 patients) or septic embolization (1 patient, cerebral embolic infarction) and were further evaluated with CT angiography or digital subtraction angiography (DSA). The undetected aneurysms were shown on CT angiography (n = 3) and DSA (n = 2). The average interval between MRI and the CT angiography or DSA was 10 days in the 5 patients.

The characteristics of the infectious aneurysms are summarized in Table 3. The size of the aneurysm varied, and the largest was measured up to 15 mm infectious aneurysminfectious aneurysm.

**Table 3:** Characteristics of infectious aneurysms.

Patient	Age	Sex	Number	Size (mm)	Location	Initial detection	Rupture	Treatment	Prognosis	Valve	Microorganism
1	20	F	> 5	3, 3, < 3	Right M3 frontal branch (3-mm) Right parietal cortical branch (3-mm) Tiny enhancing lesions in both parietal lobes	MRA	Yes	Clipping operation	Good	NV	*S. sanguinis*
2	49	F	1	9	Right M4	MRA	No	Antibiotics (vancomycin)	Good	NV	*S. anginosus*
3	25	M	1	< 3	Right M4 parietal branch	MRA	Yes	Antibiotics (vancomycin)	Good	NV	None
4	34	M	3	< 3	Right ACA, MCA, PCA terminal branch	MRA	No	Glue embolization	Good	NV	*S. parasanguinis*
5	69	F	2	< 3	Left M4 Left A5	CTA	Yes	Decompressive craniectomy	Dead	PV	*S. constellatus*
6	58	F	1	8	Right SCA distal branch	CTA	Yes	Decompressive craniectomy	Dead	NV	*S. oralis*
7	48	M	1	15	Left M4	CTA	No	Antibiotics (penicillin G)	Good	NV	*S. anginosus*
8	47	M	1	< 3	Left A5	DSA	No	Antibiotics (ceftriaxone + vancomycin)	Good	NV	None
9	67	F	2	< 3	Left PCA distal branch Right PCA distal branch (very tiny)	DSA	No	Glue embolization	Good	NV	*S. mutans*

*ACA* anterior cerebral artery, *MCA* middle cerebral artery, *PCA* posterior cerebral artery, *SCA* superior cerebellar artery, *MRA* magnetic resonance angiography, *CTA* computed tomography angiography, *DSA* digital subtraction angiography.

Notably, among the 9 patients with infectious aneurysm, aneurysmal rupture occurred in 4 (44%, 4 of 9), with a median period of aneurysmal rupture of 5 days (range 4–78 days). Of these 4 patients, 2 with aneurysms detected on MR angiography were followed-up without significant neurological sequelae after clipping surgery, whereas the other 2 patients with aneurysms that were undetected on MR angiography died despite decompressive craniectomy. Figure 2 and Supplementary Fig. e-2 show images of two patients with left-sided infective endocarditis and undetected/detected infectious aneurysms on MR angiography, respectively.

## Discussion

This study evaluated the detection rate of neuroimaging abnormalities using brain MR imaging in patients with newly diagnosed left-sided infective endocarditis. The overall detection rate of neuroimaging abnormality on brain MRI in patients with left-sided infective endocarditis was 77% (89 of 115). Moreover, the detection rate in patients with left-sided infective endocarditis without any neurological symptoms was 70% (56 of 80). In this cohort, the incidence of infectious aneurysm was 7.8% (9 of 115) whereas the detection rate on the initial screening image of MR angiography was 44.4% (4 of 9). The detection rate of neuroimaging abnormality in patients with left-sided infective endocarditis was high and was associated with poor prognosis. Thus, brain MRI might be considered in patients with suspected left-sided infective endocarditis regardless of neurological symptoms. Further evaluation, including CT and digital subtraction angiographies, should be considered as soon as possible when an infectious aneurysm is suspected.

To our knowledge, this is the largest study to evaluate the detection rate of neuroimaging abnormality in patients with left-sided infective endocarditis after additionally including a large number of patients without neurological symptoms. Previous studies and review articles have similarly evaluated the detection rate of neuroimaging abnormality on brain MRI at 65–82%^5,25–27^, which are comparable to our results. In our study, the detection rate of neuroimaging abnormality on brain MRI in patients with left-sided infective endocarditis without neurological symptoms was 70% (56 of 80; 95% CI 59–79%). The 2015 ESC guidelines have mentioned that the impact of brain MRI on infective endocarditis diagnosis is significant in patients without neurological symptoms^10^, and AHA guidelines have mentioned that brain MRI may be considered in all patients with left-sided infective endocarditis without neurological symptoms^11^. In addition, a recent study concluded that brain MRI was closely related to abnormalities that affect diagnostic or therapeutic plans in patients with infective endocarditis and emphasized the potential role of screening brain MRI regardless of neurologic symptoms. Therefore, our results are in line with these guidelines^10,11,25^ and the current studies^28^, extending evidence for performing brain MRI in patients with suspected left-sided infective endocarditis regardless of neurological symptoms.

In the present study, 9 out of 115 patients (7.8%) had infectious aneurysms, and this proportion is slightly higher than in previous studies^12,29^. Additionally, these findings revealed a low detection rate of initial MR angiography in infectious aneurysm detection. Monteleone et al.^30^ reported that the absence of hemorrhage on noninvasive imaging provided a substantial negative predictive value for the presence of an infectious aneurysm. However, owing to this condition’s rarity, widely accepted guidelines for the infectious aneurysm workup are unavailable. Specifically, five of the nine patients with infectious aneurysm had negative findings on MR angiography due to peripheral localization and small size. Even with intracranial TOF and contrast-enhanced MR angiography, the scan range and resolution remain limited. Fortunately, these five patients were confirmed to have infectious aneurysms on CT angiography or digital subtraction angiography. Moreover, the rupture rate of infectious aneurysms was 44%, with a median period of aneurysm rupture of 5 days, including two patients who died due to infectious aneurysm rupture. Although a previous review article by Cantier et al.^25^ concluded that digital subtraction angiography can only be used in patients with evidence of hemorrhage, we suggest that CT angiography or digital subtraction angiography can promptly be considered in patients with neurologic symptoms or other septic embolization including stroke, septic pulmonary embolism, splenic or renal infarction, and ischemia of the extremities, regardless of evidence of hemorrhage.

Our study supports the high prevalence of cerebral microbleeds in patients with left-sided infective endocarditis and showed that a significant number of cerebral microbleeds might have increased during follow-up. Specifically, cerebral microbleeds were detected in 57% (66 of 115) of patients with left-sided infective endocarditis, in 66% (23 of 35) of patients with neurological symptoms, and in 54% (43 of 80) of patients without neurological symptoms. Furthermore, 42% (10 of 24 patients) showed an increasing pattern among the 24 patients who received follow-up brain MRI, which were similar to a previous study showing an increasing pattern of cerebral microbleeds in 5 out of 11 patients^31^. Although the etiology and significance of cerebral microbleeds remain unclear, they are considered causal factors of infective endocarditis^32^. It has been hypothesized that cerebral microbleeds constitute markers for disease severity^26,33^. Therefore, the presence of cerebral microbleeds should not be underestimated, and require further guidelines for management.

Our study had limitations. First, as the data of consecutive patients was retrospectively collected at a single institution, the detection rate of neuroimaging abnormality could have been overestimated. Second, we did not evaluate whether brain MRI can affect clinical decision due to the retrospective design of our study. As the effectiveness of MRI on clinical decision can affect overall survival, additional studies are needed to address this. Third, there may have been confounders that could have biased the asymptomatic population to undergo brain MRI because not all neurologically asymptomatic patients with infective endocarditis undergo brain MRI. Lastly, there were a few limitations in evaluating microbleed changes, since follow-up MRIs had different magnetic field strengths or sequences as compared to previous MRI. In addition, although the susceptibility-weighted imaging (SWI) sequence is considerably more sensitive tool for the detection of microbleed, our brain MRI protocol only included gradient echo imaging. Further studies should be performed with standardized protocols including SWI sequence to assess the clinical significance of cerebral microbleeds properly.

In conclusion, the detection rate of brain MRI for neuroimaging abnormality in newly diagnosed left-sided infective endocarditis was high, and brain MRI may be considered in patients with definite or possible left-sided infective endocarditis according to the modified Duke criteria, regardless of the presence of neurological symptoms. Further evaluation, including CT angiography and digital subtraction angiography, should promptly be considered when infectious aneurysm is suspected.

### Supplementary Information

Supplementary Information.

## Data Availability

The datasets generated during and/or analyzed during the current study are available from the corresponding author on reasonable request.
