# Supplementary material for: Detection rate of brain MR and MR angiography for neuroimaging abnormality in patients with newly diagnosed left-sided infective endocarditis
Source: Sci Rep. 2023 Oct 10;13:17070. doi: 10.1038/s41598-023-44253-w (PMC10564872; doi:10.1038/s41598-023-44253-w)
Supplement: Supplementary file 1 — Supplementary Information. [file 41598_2023_44253_MOESM1_ESM.docx]

**Supplemental Materials**

Supplemental Tables

**Table e-1**. Sequence parameters for MR protocols (Ingenia; Phillips Healthcare)

| Parameter | T1WI | T2WI | T2 FLAIR | T1WI | GRE | DWI | CE 3D T1WI | 3D intracranial TOF-MRA | CE MRA |
| --- | --- | --- | --- | --- | --- | --- | --- | --- | --- |
| Sequences | 2D, SE | 2D, TSE | 2D, TSE | 2D, SE | 2D, FFE | EPI | 3D, FFE | 3D, FFE | 3D, FFE |
| TE (ms) | 10 | 80 | 125 | 10 | 15 | 60 | NA | 3.5 | 1.6 |
| TR (ms) | 450 | 3000 | 11000 (TI^*^ 2800) | 450 | 650 | 3000 | (TI^*^ 1000) | 25 | 4.3 |
| Flip angle (°) | 70 | 90 | 90 | 70 | 18 | 90 | 8 | 20 | 27 |
| FOV (mm) (RL × AP × FH)^†^ | 160 × 220 | 180 × 230 | 180 × 230 | 180 × 230 | 180 × 230 | 250 × 250 | 240 × 240 | 200 × 250 × 100 | 320 × 110 × 380 |
| Number of slices | 23 | 22 | 22 | 22 | 22 | 22 | 55 | 180 | 180 |
| Slice orientation | Sagittal | Axial | Axial | Axial | Axial | Axial | Sagittal | Axial | Coronal |
| Slice thickness (mm) | 5 | 5 | 5 | 5 | 5 | 5 | 3 | 1.2 | 1.2 |

* Inversion time. † Right/left; anterior/posterior; and foot/head. T1WI = T1-weighted image. T2WI = T2-weighted image. FLAIR = fluid-attenuated inversion recovery image. GRE = gradient echo image. DWI = diffusion weighted image. CE = Contrast-enhanced. TOF-MRA = time-of-flight magnetic resonance angiography. SE = spin echo. TSE = turbo spin echo. FFE = fast field echo. EPI = echo-planar imaging. TE = echo time. TR = repetition time. FOV = field of view

**Table e-2**. Sequence parameters for MR protocols (Architect; GE Healthcare)

| Parameter | T1WI | T2WI | T2 FLAIR | T1WI | GRE | DWI | CE 3D T1WI | 3D intracranial TOF-MRA | CE MRA |
| --- | --- | --- | --- | --- | --- | --- | --- | --- | --- |
| Sequences | 2D, TSE | 2D, TSE | 2D, TSE | 2D, TSE | 2D, SPGR | 2D, MUSE | 3D, MPRAGE | 3D, SPGR | 3D, SPGR |
| TE (ms) | 24 | 102 | 105 | 24 | 15 | Minimum | 3 | 3.4 | 1.6 |
| TR (ms) | 2300 | 4000 | 9000 (TI^*^ 2462) | 2400 | 500 | 5318 | 7 | 23 | 4.3 |
| Flip angle (°) | 111 | 142 | 160 | 111 | 20 | NA | 8 | 18 | 22 |
| FOV (mm) (RL × AP × FH)^†^ | 230 × 230 | 230 × 230 | 230 × 230 | 230 × 230 | 230 × 230 | 230 × 230 | 180 × 256 × 256 | 230 × 190 × 108 | 380 × 100 × 266 |
| Number of slices | 23 | 22 | 22 | 22 | 22 | 22 | 55 | 168 | 172 |
| Slice orientation | Sagittal | Axial | Axial | Axial | Axial | Axial | Sagittal | Axial | Coronal |
| Slice thickness (mm) | 5 | 5 | 5 | 5 | 5 | 5 | 3 | 1.2 | 1.2 |

* Inversion time. † Right/left; anterior/posterior; and foot/head. T1WI = T1-weighted image. T2WI = T2-weighted image. FLAIR = fluid-attenuated inversion recovery image. GRE = gradient echo image. DWI = diffusion weighted image. CE = Contrast-enhanced. TOF-MRA = time-of-flight magnetic resonance angiography. TSE = turbo spin echo-accelerated. MPRAGE = magnetization prepared rapid gradient echo. SPGR = spoiled gradient recalled echo. TE = echo time. TR = repetition time. FOV = field of view

**Table e-3**. Sequence parameters for MR protocols (Skyra; Simens Medical Solutions)

| Parameter | T1WI | T2WI | T2 FLAIR | T1WI | GRE | DWI | CE 3D T1WI | 3D intracranial TOF-MRA | CE MRA |
| --- | --- | --- | --- | --- | --- | --- | --- | --- | --- |
| Sequences | 2D, SE | 2D, TSE | 2D, TSE | 2D, SE | 2D, FLASH | RESOLVE | 3D, MPRAGE | 3D, FLASH | 3D, FLASH |
| TE (ms) | 7.4 | 86 | 105 | 7.4 | 16 | 70 | 3 | 21 | 3.5 |
| TR (ms) | 400 | 4000 | 9000 (TI^*^ 2500) | 450 | 450 | 4200 | 1800 | 3.7 | 1.4 |
| Flip angle (°) | 75 | 150 | 150 | 70 | 20 | 180 | 9 | 22 | 22 |
| FOV (mm) (RL × AP × FH)^†^ | 230 × 230 | 210 × 210 | 210 × 210 | 210 × 210 | 210 × 210 | 210 × 210 | 370 × 108 × 256 | 220 ×198 × 108 | 370 × 108 × 277 |
| Number of slices | 20 | 20 | 20 | 20 | 20 | 20 | 50 | 180 | 180 |
| Slice orientation | Sagittal | Axial | Axial | Axial | Axial | Axial | Sagittal | Axial | Coronal |
| Slice thickness (mm) | 5 | 5 | 5 | 5 | 5 | 5 | 3 | 0.5 | 0.6 |

* Inversion time. † Right/left; anterior/posterior; and foot/head. T1WI = T1-weighted image. T2WI = T2-weighted image. FLAIR = fluid-attenuated inversion recovery image. GRE = gradient echo image. DWI = diffusion weighted image. CE = Contrast-enhanced. TOF-MRA = time-of-flight magnetic resonance angiography. SE = spin echo. TSE = turbo spin echo. FLASH = fast low angle shot. MPRAGE = magnetization prepared rapid gradient echo. TE = echo time. TR = repetition time. FOV = field of view

Supplemental Figures


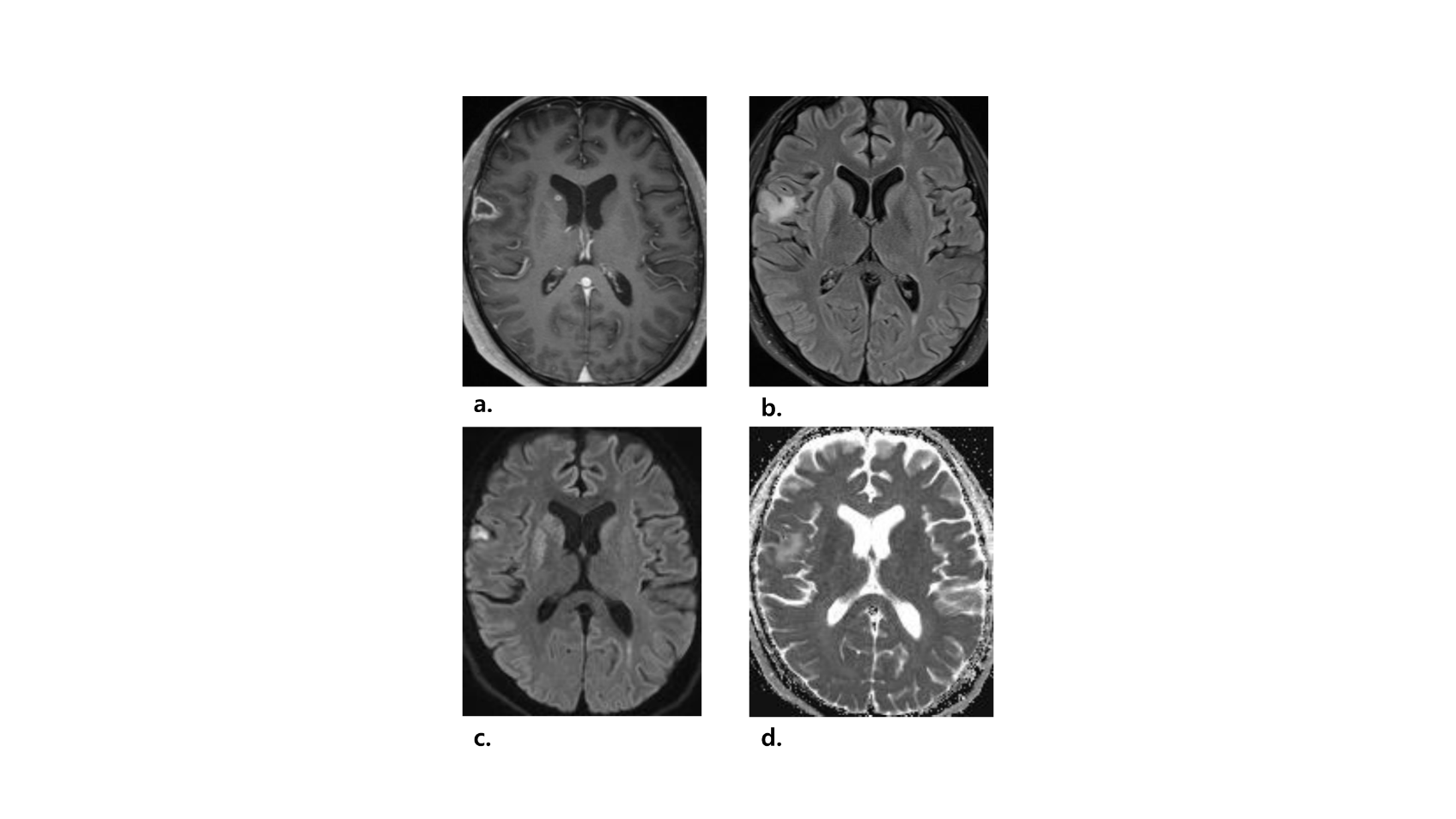


**Figure e-1.** Images of a 27-year-old man with fever and drowsy mentality. Two small rim-enhancing lesions in the right frontal lobe and right basal ganglia were visualized on **(a)** contrast-enhanced T1-weighted imaging. The larger lesion shows hyperintense peripheral edema on **(b)** fluid-attenuated inversion recovery imaging and central diffusion restriction on **(c, d)** diffusion-weighted imaging that suggested an abscess. Left-sided infective endocarditis was confirmed after echocardiography and blood culture.

**
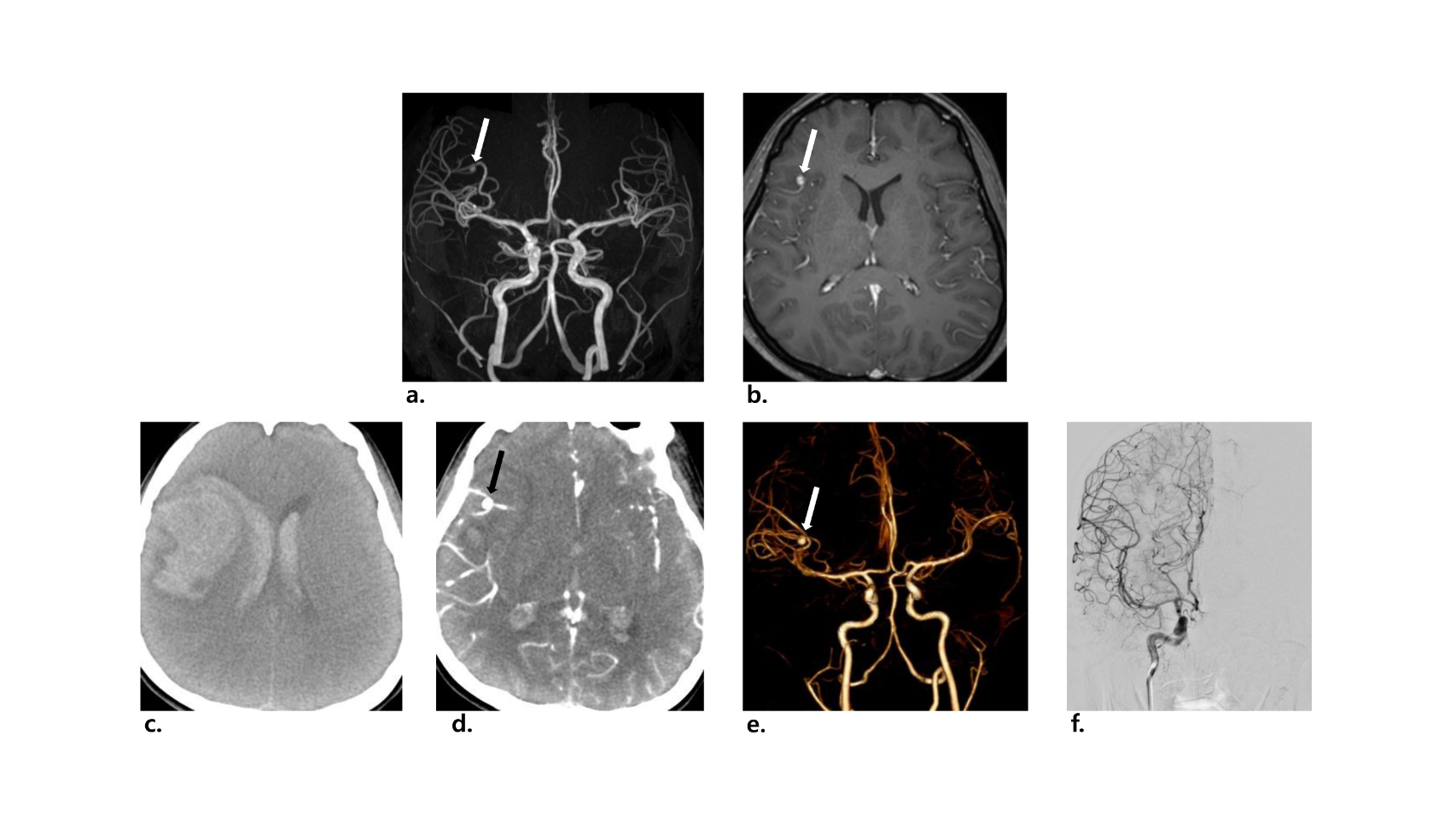
**

**Figure e-2.** Images of a 20-year-old woman with left-sided infective endocarditis. **(a, b)** Brain MRI and MR angiography revealed a 3-mm mycotic aneurysm (arrows in **a** and **b**) at the superior branching of the right MCA (right M3 frontal branch) on **(a)** intracranial 3D time-of-flight (TOF) MR angiography and **(b)** contrast-enhanced T1-weighted imaging. After 3 days, the patient developed left hemiparesis and drowsy mentality. **(c)** Axial non-contrast-enhanced CT imaging shows a new acute intracranial hemorrhage in the right frontotemporal lobe, along with diffuse intraventricular hemorrhage. **(d, e)** Images from contrast-enhanced CT and CT angiography show an increase in the size of the mycotic aneurysm (arrows in **d** and **e**) at the right M3 frontal branch (3 mm 🡪 5 mm), suggesting that a mycotic aneurysmal rupture at the right M3 frontal branch caused the acute intracranial and intraventricular hemorrhages. After emergency clipping operation and hematoma evacuation, **(f)** the mycotic aneurysm was no longer visible on follow-up transfemoral cerebral angiography.
